# Towards a safety net for management of 22q11.2 deletion syndrome: guidelines for our times

**DOI:** 10.1007/s00431-013-2240-z

**Published:** 2014-01-03

**Authors:** Alex Habel, Richard Herriot, Dinakantha Kumararatne, Jeremy Allgrove, Kate Baker, Helen Baxendale, Frances Bu’Lock, Helen Firth, Andrew Gennery, Anthony Holland, Claire Illingworth, Nigel Mercer, Merel Pannebakker, Andrew Parry, Anne Roberts, Beverly Tsai-Goodman

**Affiliations:** 1 North Thames Regional Cleft Unit, Great Ormond Street NHS Trust, Great Ormond Street, London, WC1N 3JH UK; 2 Pathology Department, Aberdeen Royal Infirmary, Foresterhill, Aberdeen, AB25 2ZD Scotland, UK; 3 Department of Clinical Immunology, Addenbrooke’s Hospital, Box 109, Cambridge, CB2 2QQ UK; 4 Royal London Children’s Hospital, Barts Health NHS Trust, Whitechapel, London, E1 1BB UK; 5 Department of Medical Genetics, Addenbrooke’s Hospital, Box 134, Cambridge, CB2 0QQ UK; 6 Papworth Hospital NHS Foundation Trust, Papworth Everard, Cambridge, CB23 3RE UK; 7 Congenital and Paediatric Cardiology Service, Glenfield Hospital, Groby Road, Leicester, LE3 9QP UK; 8 Department of Medical Genetics, Cambridge University Hospitals Foundation Trust, Cambridge, CB2 2QQ UK; 9 Old Children’s Outpatients, Great North Children’s Hospital, Royal Victoria Infirmary, Queen Victoria Road, Newcastle-upon-Tyne, NE1 4LP UK; 10 Section of Developmental Psychiatry, University of Cambridge, 2nd Floor, Douglas House, 18b Trumpington Street, Cambridge, CB2 8AH UK; 11 East of England Cleft Network, Addenbrooke’s Hospital, Box 46, Cambridge, CB2 2QQ UK; 12 Cleft Unit of the South West of England, Frenchay Hospital, Bristol, BS16 1LE UK; 13 Primary Care Unit, Department of Public Health and Primary Care, University of Cambridge, Strangeways Research Laboratory, Worts Causeway, Cambridge, CB1 8RN UK; 14 Cardiac Centre, Bristol Royal Hospital for Children, Paul O’Gorman Building, Upper Maudlin Street, Bristol, BS2 8BJ UK; 15 South West Cleft Unit, North Bristol NHS Trust, Beckspool Road, Bristol, BS16 1JE UK

**Keywords:** 22q11 deletion syndrome, Guidelines, Di George, Velocardiofacial, Congenital abnormalities, Resource management

## Abstract

The commonest autosomal deletion, 22q11.2 deletion syndrome (22q11DS) is a multisystem disorder varying greatly in severity and age of identification between affected individuals. Holistic care is best served by a multidisciplinary team, with an anticipatory approach. Priorities tend to change with age, from feeding difficulties, infections and surgery of congenital abnormalities particularly of the heart and velopharynx in infancy and early childhood to longer-term communication, learning, behavioural and mental health difficulties best served by evaluation at intervals to consider and initiate management. Regular monitoring of growth, endocrine status, haematological and immune function to enable early intervention helps in maintaining health. *Conclusion*: Guidelines to best practice management of 22q11DS based on a literature review and consensus have been developed by a national group of professionals with consideration of the limitations of available medical and educational resources.

## Introduction

The effective and efficient use of resources in conditions with multiple disabilities is one of today’s major challenges. A United Kingdom (UK) government-led initiative, the National Service Framework for Children [11], aims for the child with complex needs to receive co-ordinated high quality child and family centred care. Needs are to be assessed through the use of evidence-based guidelines and protocols which are regularly updated, and their implementation subject to local audit. The present international economic climate is affecting many national health budgets adversely [14, 64]. It is therefore timely to consider the minimum set of safe medical standards and screening procedures for 22q11.2 deletion syndrome (22q11DS) consistent with best practice recommended internationally [5, 19]. In addition to identifying established problems such standards should also anticipate their onset whenever possible. This has the benefit of potentially avoiding or reducing complications likely to impair well-being and additional burdens on health and community resources.

The 22q11DS population prevalence is reported to be one in 4,000 to one in 6,000 [6]. It equates annually to between 900 and 1,350 affected individuals from among the 5.4 million births each year in the European Union. With present-day treatment, survival beyond infancy is 90–95 % [32, 50], although life span may be reduced in some as adults [4]. As they become adults the less severely affected may become parents, their children adding to the burden of care for the community. The number of affected individuals of all ages in the UK and Ireland, population 66 million, is probably 10,000–15,000, assuming survival to middle age. It is likely that only the more severely affected children and a small proportion of adults are currently correctly identified and receiving appropriate support from social, educational and health services [33].

**Fig. 1:**
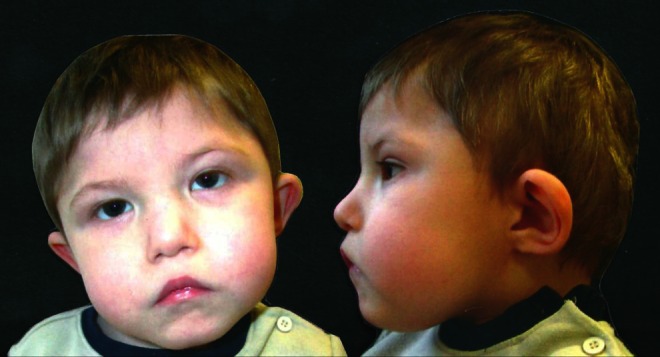
Child with 22q11.2 deletion syndrome

The 22q11.2 deletion is a 1.5- to 3-megabase deletion on the long (q) arm of chromosome 22. The deletion contains TBX1, the major candidate gene, and other genes, controlling the third and fourth pharyngeal arches, brain and skeletal development. Haploinsufficiency results in the principal syndrome phenotype. No correlation between the size or site of the deletion with phenotype has yet been found [22, 29, 39]. The deletion occurs spontaneously in 85–90 % of patients or is inherited from either parent in an autosomal dominant fashion. An unaffected parent may carry the deletion in their egg or sperm (germline mosaicism); their recurrence risk is 1 % [51]. The syndrome is a multisystem disorder with several major features, and many less severe abnormalities which aid detection [59], in conjunction with typical facial dysmorphia (Fig. 1) which collectively are not always appreciated by clinicians. The multitude of combinations can cause diagnostic confusion, a legacy of which has been the nomenclature applied to apparently different syndromes (Di George, velocardiofacial, conotruncal) now known to be usually due to the 22q11.2 deletion [67]. Those with the phenotype but without the deletion comprise up to 20 % and are usually referred to as Di George syndrome; their management follows the same guidelines. Rates of detection increase where specialists are familiar with the condition [46]. Diagnosis in 95 % of subjects with the deletion has been by fluorescent in situ hybridisation (FISH), and is being superseded by methods that also reveal the 5 % of atypical deletions which FISH fails to identify. They include array comparative genome hybridisation (aCGH), genome wide microarrays and multiplex ligation-dependent probe amplification (MPLA) [40].

The major conditions occurring in approximately 70 % or more are congenital heart disease, immune deficiency, palate defects affecting feeding and speech, and learning difficulties [28]. Those found in 25–50 % include feeding disorders, early growth faltering, gut dysmotility, psychiatric, behavioural and neurological conditions, structural (renal, skeletal, brain, gastrointestinal, eye and dental) abnormalities, hearing impairment, hypocalcaemia, haematological and autoimmune disorders [32, 34, 50, 61].

## Method

Guidelines for the management of 22q11DS evolved from a consensus document initiated by the parent-led charity Max Appeal [31], to promote good practice in the UK across publicly funded community and health services. The consensus participants were parents, clinicians and therapists with extensive experience in managing the condition.

The SIGN [52] systematic literature review system’s highest level of evidence is 1++ through eight grades to the lowest level 4; it was applied to the 22q11DS literature found in PubMed and via the National Health System Athens search engine up to December 2012. It comprised some case control studies, many case series and numerous case reports. Those reported here by level in {} brackets ranged from {2+} to {4}. The highest grade of recommendation is {A} though {D}. Those relating to 22q11DS generally comprised grade D {D} defined as evidence level {3} or {4} or extrapolated evidence from studies rated as {2+}, and Good Practice {GP} recommendations based on the clinical experience of the guideline development group. By extrapolation, conventional treatment appears as efficacious in 22q11DS as for those with similar but unrelated conditions, with a significant exception being velopharyngeal disproportion (VPD), and informed the recommendations accordingly.

Age appropriate investigations and assessments were evaluated from the literature, the prevalence of 22q11DS associated disorders, and the need for information to guide management and advice giving [28, 29, 32, 34, 50, 61].

## Presentations

### By age

Fetal anomaly screening may result in identifying that the foetus is affected, and in some instances one of the parents, more likely the mother, is also affected. Careful multidisciplinary assessment of the pregnancy is required {C}.

**Fig. 2:**
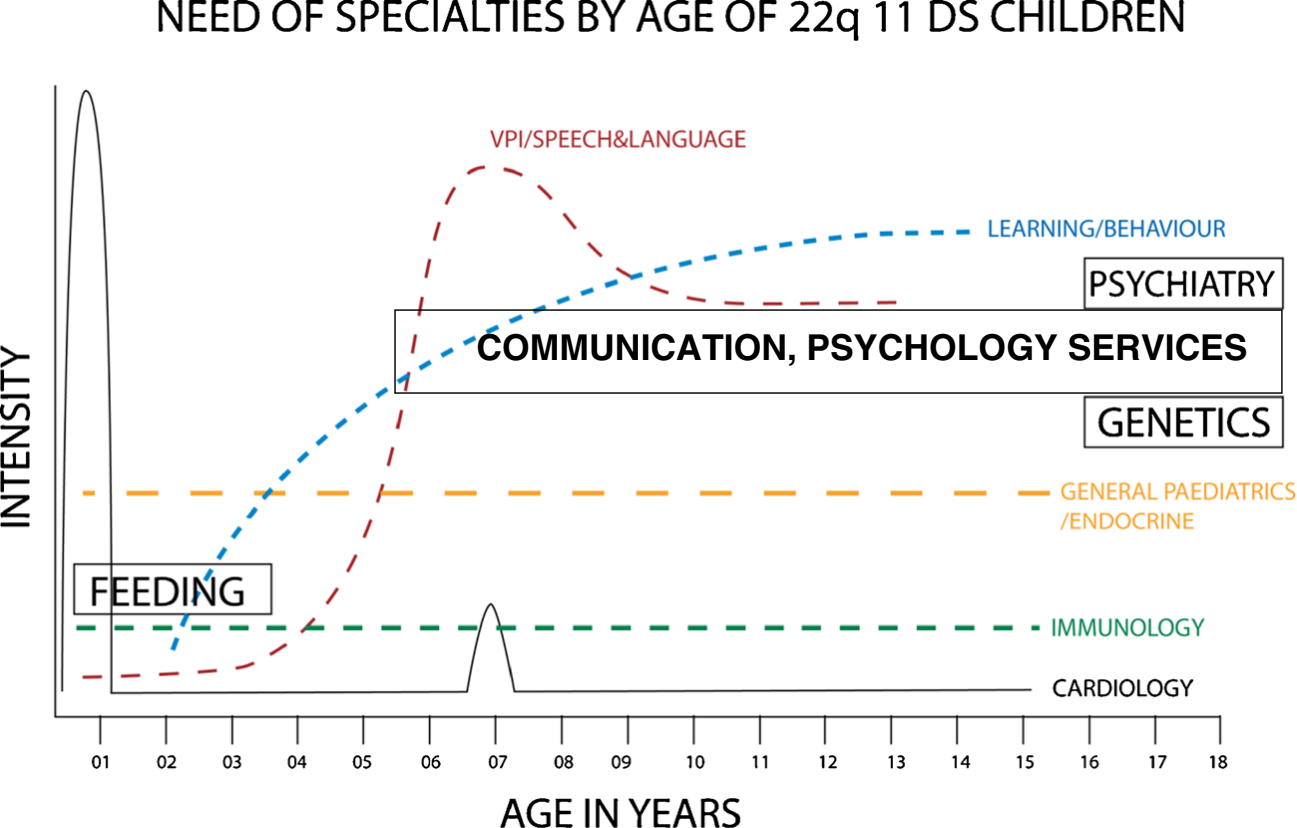
Need of specialties by age of 22q11 DS children

The individual with 22q11DS is likely to present changing clinical and psychosocial priorities from birth to maturity (Fig. 2). Early care is dominated by organ malformations requiring surgery, feeding support, and treating infections; as childhood progresses, neurodevelopmental, behavioural and educational priorities require attention; in adolescence, scoliosis monitoring with possibly surgical intervention, and psychosocial support; in adults socioeconomic, general medical and psychiatric support.

Severity, even between affected members of the same family, is highly variable {3}.

### By system

#### Gestalt

Facial dysmorphia (Fig. 1) are subtle especially in infancy, and also appear less marked in adults. They include long narrow face, almond shaped eyes, a bulbous nose (becoming evident with age), small mouth, overfolded ear helix, asymmetry of facial movement {3}, and occasionally skull asymmetry due to craniosynostosis {4}.

#### Cardiac disorders

Cardiac malformation prevalence varies from 80 % to 92 % of those presenting in infancy [43, 46] to 40–50 % presenting in childhood [29, 35], and predominantly comprise conotruncal anomalies. They may appear shortly after birth with cyanosis due to reduced blood flow to the pulmonary circulation by right ventricular outflow obstruction as in Fallot’s tetralogy and pulmonary atresia, (with or without multiple aortopulmonary collateral arteries [MACPA]), or with cardiovascular collapse due to systemic outflow obstruction from aortic arch narrowing or interrupted aortic arch (most frequently type B) [32, 50]. The obverse, an increase in blood flow due to large shunts such as VSD (often perimembranous), and truncus arteriosus, may present with heart failure within a few days or weeks. An aberrant subclavian artery may present with feeding difficulties or respiratory symptoms. Alternatively, conditions such as right sided aortic arch or valve defects may be identified only incidentally after initial referral for concern about other issues, for example, about speech or developmental delay.

Management: a cardiology opinion, electrocardiogram and echocardiogram are mandated at the time of diagnosis of the deletion if not performed before {C}. Treatment is individualised according to the underlying lesion. If the first assessment is normal no further routine review is required.

#### Hypocalcaemia

Hypocalcaemia is usually due to hypoparathyroidism, variable in duration (from transient in neonates, to resolving in months to years) and is rarely lifelong. Point prevalence of hypocalcaemia outside the neonatal period was 30 % of 27 subjects of all ages [58], with a lifetime prevalence of 50 % [3]. Hypocalcaemia was detected in 40 % of those with seizures [19, 50]. It presents clinically as jitteriness, seizures which may need to be further investigated as prevalence of epilepsy is increased, or stridor that needs to be differentiated from laryngeal web which is also more prevalent in 22q11DS. Symptomatic or biochemical hypocalcaemia may also be precipitated by the stress of surgery, and by increased needs during puberty or pregnancy. Dentition may be prone to caries {4}.

Management: monitor by checking the (ionised) calcium level 3 monthly in infancy and annually thereafter {D}. Low calcium and raised phosphate should initiate a check for an inappropriately low parathyroid hormone and normal vitamin D level.

Calcium supplements and various formulations of vitamin D and analogues are generally effective treatments {C} but careful monitoring is required in patients with associated structural renal anomalies such as unilateral renal agenesis or dysplastic kidney. Daily Vitamin D is advised for all ages {GP}. The dose should be the recommended daily allowance, or as therapeutically indicated {B}.

#### Immune system disorders

(a) Immune disorders affect the majority of patients, are commonly mild and manifest mainly as reduced T cell numbers and function. The thymus may be absent. Reduced levels of IgA and IgM in older children occur more frequently than the general population. The T-cell deficiency undergoes spontaneous improvement in most individuals, usually by 2 years of age.

Almost all patients have a greater than average tendency to infections, particularly of the respiratory tract, even when laboratory tests of immune function appear normal [16]. Pneumonia occurs in 10 %, mainly in infancy and preschool. Recurrent upper respiratory infections, especially otitis media, are more frequent and persistent; concomitant VPD may contribute. Infectious episodes reduce in frequency with age. Early feeding problems may result in nutritional anaemia; autoimmune (Evans) anaemia and significant thrombocytopenia may occur at any age.

Monitor: baseline immunology testing (see Table 1) {C}, and annual full blood count {GP}.

**Table 1:** Recommendations for investigation, management and referral

Investigations at diagnosis
• At diagnosis
a. Full blood count including differential white cell count, lymphocyte phenotyping, immunoglobulins G, A, M. Lymphocyte proliferation testing if readily available and T cell count low; post immunisation tetanus or Hib antibodies {B}. b. Serum calcium, thyroid function {B}. c. Cardiological examination, echocardiogram and electrocardiogram {B}. d. Parental 22q11 status, and siblings if a parent is affected {B}. e. Renal ultrasound looking for single kidney, cysts, dilated collecting system {B}.
Essential initial actions
• Irradiated cytomegalovirus negative blood products if immune status is unknown or severely affected {C}. Urgent specialist referral if T lymphocytes appear virtually absent or very low.
• Vaccination: Primary vaccination should be given promptly, including Mumps Measles and Rubella (MMR) {D} even if the CD4 count is low {D}. Chickenpox vaccination is not given with a CD4 count below 200/ml, as in Human Immunodeficiency Virus {GP}. Avoid BCG, and consult an immunologist if circumstances require {GP}.
Specific medical examinations
• Genetics: At diagnosis, and repeated when the family and emerging adult have a need {GP}.
• Special senses: hearing test and eye examination at diagnosis {C}. Orthoptic and refractive examination at 3 years, and ophthalmic examination as clinically indicated.
• Musculoskeletal system: Scoliosis examination at diagnosis and between 10 and 12 years, in the earlier part of the adolescent growth spurt {D}. Locomotor system examination for limb pain, stiffness, swelling; arthritis can present as delayed development in young children. Depending on clinical findings appropriate investigations include inflammatory markers, autoimmune serology, and ultrasound {GP}.
• Monitor height and weight frequently up to 2 years old, annually thereafter. Slowing of growth warrants full assessment, including screening for thyroid and growth hormone deficiency {D}.
• Autoimmune disorders if clinically indicated: autoantibodies including direct antiglobulin test and thyroid antibodies {D}.

Management: only 1 % have no T cells, requiring urgent referral to a supra-regional centre for assessment and, if available, thymus transplant {C}. Antifungal, antiviral and anti-pneumocystis prophylaxis and immunoglobulin replacement therapy should be commenced {B, C}. Cytomegalovirus negative and irradiated blood products are not routinely administered to infants undergoing cardiac surgery in the UK unless an immune deficient state is suspected {C}.

In selected individuals, who present with recurrent upper respiratory tract infection, even with ‘normal’ immune function tests, antibiotic prophylaxis in winter may be beneficial in reducing infections and supporting school attendance {GP}.

(b) Autoimmune phenomena. Auto-immune conditions are found in up to a third, and symptomatic in a fifth as juvenile chronic arthritis (JIA), autoimmune thrombocytopenia, haemolytic anaemia, Raynaud’s phenomena, and autoimmune thyroid disease [9, 16, 27]. Low IgA is a feature in JIA in 22q11DS [9].

Monitor: clinical review and annual full blood count {D}.

#### Feeding and growth

(a) Feeding problems. Feeding problems in the first year are common [50], affecting up to 68 % [29]. Cleft palate and submucous cleft palate (SMCP) are found in 14 % [50]. If feeding difficulties are encountered in the neonatal period, the possibility of an overt or a SMCP must be investigated and the child referred to the local specialist cleft lip and palate service who will advise on feeding and whether a modified cleft feeding bottle may be appropriate.

Gastro-oesophageal reflux is common and dysphagia (10 %) may cause recurrent silent aspiration and pneumonia.

Management includes thickeners, anti-reflux and propulsive medication {C}, and nasogastric feeding. Gastrostomy is indicated if the airway is significantly compromised, as demonstrated on X-ray barium contrast swallow or associated with recurrent aspiration pneumonia {C}.

(b) Growth disorders. Weight faltering frequently occurs {2+}. Although 40 % fall below the 3rd centile in height and weight in the first year [23, 50], this does not correlate with cardiac or feeding disorders. Catch-up in height takes place by late childhood ultimately to a little below average by adult life, with a prevalence of obesity observed in French children [47] to be similar to the surrounding general population {3}. Hypothyroidism, hyperthyroidism and growth hormone deficiency are increased in frequency {4}.

Management: see Table 1.

#### Genitourinary tract

Renal abnormalities are found in 7–36 %, comprising absent, dysplastic or multicystic kidneys, obstructive abnormalities, vesicoureteric reflux, and nephrocalcinosis secondary to calcium and vitamin D supplementation [29, 32, 50]. Genital anomalies including undescended testes and hypospadias are increased.

Management: ultrasound examination generally determines the pathway for management {B}.

#### Musculoskeletal system disorders

(a) Hypotonia and ligamentous laxity are very common, as is a disinclination to walk even short distances, with frequent complaint of leg pains which are usually symmetric; asymmetry suggests other pathologies {3}. The pain may cause waking at night, contributing to daytime inattention. Unsteady gait, incoordination and clumsy hand skills are common. Patella dislocation and rheumatoid arthritis are increased [9] compared with the general population {4}.

Management: assess gait, limb pain, joint swelling, early morning stiffness, or delayed motor development with these comorbidities in mind. Specialist referral to a paediatric rheumatologist and specialist physiotherapist may be indicated {GP}.

(b) Cervical spine examination. Radiological abnormalities of the cervical spine region are very common [48]. As in Down’s syndrome and other genetic syndromes [36] evidence of cord compression is rare {3}, and evidence is lacking that cervical X-rays have validated predictive value for subsequent acute dislocation/subluxation at the atlantoaxial joint [30, 65].

Management: seek advice expeditiously in the presence of any physical or neurological symptoms or signs of cord compression. Anaesthetists should be alerted to the possibility of cervical spine injury whilst manipulating the head and neck in the unconscious 22q11DS patient. No limitations on sports are imposed, subject to suitable supervision and support {GP}.

(c) Scoliosis. The prevalence of scoliosis in 22q11DS is 9–24 % [8, 29, 46, 61], 3–8 times that of the general population. Of those with scoliosis 6 % require surgical management [8]. Most frequently the radiological appearance is of Adolescent Idiopathic Scoliosis. Hemivertebrae and other skeletal anomalies also occur.

Management: clinical examination of the spine at diagnosis and ages 10 to12 years is recommended {D}.

#### Speech and communication disorders

Articulation and communication problems occur in two-thirds, characterised by hypernasal articulation due to VPD [32], cleft palate and SMCP. Expressive speech delay may initially be greater than performance based abilities, yet subsequently overtake the latter [54]. This phenomenon is considered to be syndrome specific [3]. Management: assessment and intervention through speech and language therapists, with early referral to a cleft surgery centre when VPD is suspected. Palatopharyngeal surgery generally improves comprehensibility, but residual VPD may remain [37, 55].

#### Hearing

Deafness is due to otitis media and secretory otitis media in 75 % {3}; up to 15 % [12, 50] have sensorineural deafness {3}. Eustachian tube dysfunction associated with cleft palate or SMCP and immune deficiency, contributes to conductive loss.

Management: universal hearing testing occurs at birth in many economically developed countries, and is repeated at 4 to 5 years {B}. Additional examinations are as indicated clinically, in the presence of delayed speech and language development {C}.

#### Intelligence and educational assessment

Intelligence scores are shifted lower. The mean IQ is in the low 70s; 30 % lie in the low normal IQ range between 80 and 100 [46, 56, 68]. Less than 20 % have moderate to severe learning difficulties. By school age verbal ability usually advances to be similar to or better than performance-related ability. Memory, and hence rote learning, is usually a strength. Ability to grasp abstract concepts, especially mathematics, is weak. Attention difficulties, visual spatial difficulties, and impaired executive function with similarities to a non-verbal learning disorder are often present {3} [10, 26, 68].

Management: it is exceptional not to receive some learning support or a special needs teacher for reading comprehension and mathematics [29, 46]. Key ages for assessment are around transitional stages in the children’s school career. In the UK this coincides with the start of primary education at 5 years, prior to secondary school at 11 years, and at 16 years, preparatory to leaving school or considering tertiary education. A staged approach, assessed by classroom teachers in concert with special educational needs co-ordinators have an important role in highlighting needs. They, in turn, are best served by informed educational psychologists, supported by clinical psychologists knowledgeable about the learning profile of these children. Some individuals may show a decline in cognitive abilities [13]. The need for formal educational assessment therefore depends on perceived severity, and availability of resources {GP}.

#### Mental health

In common with other neurogenetic syndromes, emotional and behavioural issues and psychiatric disorders occur more frequently than in the general childhood population, affecting 20–50 % of 22q11DS individuals at any age. Often co-occurring, problems can include attention deficit hyperactivity disorder (ADHD), obsessive compulsive disorder, depression, anxiety disorders and autistic spectrum disorder (ASD) {2+} [1, 18] to {3} [63]. Children often show rapid mood changes and atypical social interaction skills, which may benefit from assessment and intervention even if formal criteria for a psychiatric diagnosis are not met {3} [53].

During adolescence and adulthood, risk of significant and persistent mental health difficulties is increased relative to comparable populations with mild to moderate intellectual disability, and mood disorders become increasingly common {3} [2, 17]. The prevalence of schizophrenia was 24 % in one adult study {3} [45], and 22.5 % in another [15]. In addition, many adults with 22q11DS suffer from depression or generalised anxiety disorder [15]. Links between childhood adjustment and later psychiatric illness in 22q11DS remain uncertain, with no single symptom or diagnosis (e.g., ADHD, ASD) strongly predicting vulnerability to schizophrenia [1, 62]. Deterioration in cognitive function, in mood and social interaction, or worsening psychotic symptoms, are all potential indicators of risk warranting early psychiatric assessment (Table 2) [17, 25].

**Table 2:** Therapeutic, psychological, behavioural and educational assessments for early intervention {D}

• Early recognition of speech difficulties and speech therapy intervention may reduce the emergence of deviant articulation. Syndrome-specific leaflets are available [21].
• Adenoidectomy may worsen articulation and should only be contemplated after expert speech assessment.
• Prompt referral for developmental monitoring involving assessment for physiotherapy, and occupational therapy according to need.
• An eye test for squints and refractive errors at 3 years.
• Child and adolescent psychological and mental health services referral for assessment when ASD, ADHD, emotional and behavioural issues in the preschool and school age child cause distress or disruption. Early psychotic symptoms should be assessed.
• Not all children will require a full educational assessment. This may be evident in the preschool period or not raise concern until adolescence when abstract concepts are increasingly a part of the curriculum. With parental approval, inform the school of the condition and potential needs. Explanatory syndrome-specific leaflet suitable for teachers can be useful [21].

Many young adults experience social isolation and employment difficulties and continue to be prone to the emergence of 22q11DS-related conditions.

Management: At all ages ready access to a psychologist or psychiatrist is advantageous {GP}. The outcome of the assessment determines the intervention (Table 3). When someone is diagnosed with a psychiatric disorder, medication might be considered (exercising caution in relation to medical co-morbidities). In case of behavioural problems, a psychological intervention could be beneficial.

**Table 3:** Regular assessments for all individuals

• Annual
a. Full blood count for cytopaenias (of red cells, white cells and platelets), serum calcium and thyroid function. b. Height and weight. c. Clinically monitor for autoimmune disease; perform autoantibody testing as indicated. d. Enquire about social–educational progress and psychological well-being.
• Regular dental care.

## Discussion

In drawing up guidelines, it was deemed appropriate to apply nationally accepted levels of clinical risk which are evidence based. These can be adapted to suit local circumstances and legal jurisdictions, for example where defensive medicine plays a significant role in clinical practice [5]. Examples of such controversy between specialists in different countries include cervical X-rays for instability [5, 30, 48], and the practice of palpation or MRI for presurgical location of carotid artery anatomy [37, 57]. In the UK, consensus against routine cervical X-rays has been agreed, but each surgical unit continues their own individual policy on obtaining a presurgical MRI to exclude medial displacement of the carotid arteries. It is impractical to be prescriptive in guidelines for management where consensus may be lacking, such as encouraging use of a signing system in early communication [41, 54] in the context of characteristic speech and language delays; some anecdotally see this as a distraction from focusing on oral communication [20].

Child advocacy is valuable in promoting understanding of the changing educational profile from among the 15 million children in the EU with special educational needs [49, 64]. In Europe, each country’s special needs education is defined and managed separately [38] and parental preference may not be taken into account [49, 64], though all countries share the common goal of an inclusive system which integrates those with disabilities and vulnerabilities into main stream schooling as far as possible. Some jurisdictions have an appeals system should the educational authorities fail to initiate the process or provide appropriate resources. In the UK, this is the First-tier Tribunal [24]. Although the European Convention on Human Rights Article 1 Protocol 2 enshrines the right of all children to a suitable education it does not guarantee it be of a suitable quality; this remains dependent on each country’s priorities and resources.

The 22q11DS UK Guidelines group, by setting what it perceives as minimum recommendations and not optimal ones as advocated in other recent guidelines [5], aims to emphasise the conserving of resources. They chime with the WHO observation of the wide variation in European countries’ response to the economic downturn, and its appeals to ‘viewing fiscal balance as a constraint to be respected…towards an emphasis on maximising the health system’s performance’ [38, 66]. The European Commission elaborates further on health services sustainability, citing the need for reforms in health budgets [14, 42]. The inception of minimum guidelines should not imply rationing of core services to this group of patients, whose needs must be balanced against possible ‘false savings’ leading to increased costs in the long term.

Advocacy (Table 4) is highlighted as a driver for adherence to the guidelines throughout the life cycle. Families with an adult 22q11DS parent are particularly vulnerable. A member of the multidisciplinary team, familiar with the condition, often Paediatrician, Clinical Geneticist, less commonly Psychiatrist or Psychologist, may be identified as ‘key worker’ in liaising with the various health, education and social services agencies. Developing a structure for transition from paediatric to adult care for individuals with learning disability and multiple comorbidities is a work in progress. The often fragmented services for adolescents and young adults with 22q11DS need to be brought together, using a model such as The Netherlands transmural care [60].The persistence of comorbidities and emergence of new conditions in adulthood [3, 4, 7] require guidelines which are continuous and flexible throughout life.

**Table 4:** Advocacy {GP}

• Social work and adult learning difficulty team referral where an affected parent or the family are in need of support and advocacy.
• Coordinated care by a key worker. An informed primary care physician or community paediatrician, and later the adult mental health team, may be best placed to holistically guide the individual’s progress.
• Developing local expertise. The multisystem nature of 22q11DS has led to a variety of specialities taking the lead in developing 22q11DS services for their country and local geographical area. For instance, psychiatry in Canada, Israel and Switzerland, cardiology in Italy and Japan, genetics in Belgium, France and parts of the US, cleft surgery centres in the US and UK.
• 22q11DS Personal Health Record booklet [44] is an example of a patient held record of clinic attendances and list of prompts for assessments to be performed.
• Managing Transitional Care from paediatric specialist to adult clinics. Planning is required. Although congenital heart often has an established pathway, holistic care for the young adult with 22q11DS has not. The need for continued contact with health services and monitoring as for younger individuals should be recognised and organised.

## Conclusions

A consensus comprehensive care plan is presented. An acceptable standard of care for individuals with 22q11DS requires careful coordination and a multidisciplinary team approach, with access to services throughout the life cycle.

## Abbreviations

22q11DS: 22q11.2 deletion syndrome
SMCP: Submucous cleft palate
VPD: Velopharyngeal disproportion
